# Genetics and Genomics of Pseudoexfoliation Syndrome/Glaucoma

**DOI:** 10.4103/0974-9233.75882

**Published:** 2011

**Authors:** Ursula Schlötzer-Schrehardt

**Affiliations:** Department of Ophthalmology, University of Erlangen-Nürnberg, Erlangen, Germany

**Keywords:** Complex Genetics, *LOXL1*, Pseudoexfoliation Syndrome, Pseudoexfoliation Glaucoma

## Abstract

Pseudoexfoliation (PEX) syndrome, one of the most common causes of glaucoma, represents a complex, multifactorial, late-onset disease of worldwide significance. The etiopathogenesis involves both genetic and non-genetic factors. The PEX-specific tissue alterations are caused by a generalized fibrotic matrix process, which has been characterized as a stress-induced elastosis associated with the excessive production and abnormal cross-linking of elastic microfibrils into fibrillar PEX aggregates. The identification of lysyl oxidase-like 1 (*LOXL1*) as a major genetic risk factor for PEX syndrome and PEX glaucoma further supports a role of elastogenesis and elastosis in the pathophysiology of PEX, as *LOXL1* is a pivotal cross-linking enzyme in elastic fiber formation and stabilization. The available data suggest that *LOXL1* is markedly dysregulated depending on the stage of the fibrotic process. While transient upregulation of *LOXL1* during the early stages of PEX fibrogenesis participates in the formation and aggregation of abnormal PEX fiber deposits, the decreased expression of *LOXL1* during the advanced stages of the disease may affect elastin metabolism and promote elastotic processes, e.g. in the lamina cribrosa, predisposing to glaucoma development. However, in view of the low penetrance of the PEX-associated risk variants of *LOXL1*, other genetic and/or environmental factors must contribute to the risk of developing the PEX phenotype. Some evidence exists for the contribution of additional genes with relatively small effects, e.g. clusterin (*CLU*), contactin-associated protein-like 2 (*CNTNAP2*), apolipoprotein E (*APOE*), glutathione S-transferases (*GSTs*), and tumor necrosis factor-alpha (*TNFA*), in certain study populations. Several environmental conditions associated with PEX, such as oxidative stress as well as pro-fibrotic cytokines and growth factors, can regulate expression of *LOXL1* and elastic proteins *in vitro* and may therefore act as co-modulating external factors. Ultimately, both detection and functional characterization of yet unidentified genetic and non-genetic factors may lead to the development of more precise screening tools for the risk of PEX glaucoma.

## INTRODUCTION

Pseudoexfoliation (PEX) syndrome is a common, age-related, systemic disorder of worldwide significance with an estimated prevalence ranging from 10% to 20% of the general population over 60 years of age.^1^ It is clinically diagnosed by observation of whitish flake-like deposits of PEX material on anterior segment structures, particularly on the anterior lens surface and the pupillary border of the iris. Despite its worldwide distribution, there is a clear tendency for PEX syndrome to cluster geographically and in certain racial or ethnic subgroups. For example, there is a high prevalence of PEX syndrome in Nordic, Baltic, Mediterranean, and Arabian populations, where it affects up to 30% of individuals over age 60. The underlying causes of the differences in prevalence rates between age-matched geographical and ethnic populations remain unknown, but appear to be mainly related to variation in genetic background.

PEX syndrome is one of the most common causes of glaucoma worldwide.^2^ PEX-associated open-angle glaucoma may account for 20-60% of open-angle glaucoma and may even show a higher frequency than the primary form of the disease in some countries such as Oman.^3^ Compared to primary open-angle glaucoma (POAG), PEX glaucoma has a more serious clinical course and worse prognosis. It is typically associated with higher mean levels of intraocular pressure (IOP), greater diurnal pressure fluctuations, marked pressure spikes, higher frequency and severity of optic nerve damage, more rapid visual field loss, poorer response to medications, and more frequent surgical intervention.^4^ In the early manifest glaucoma trial, the presence of PEX was the most important independent risk factor for glaucoma progression.^5^ Apart from glaucoma development, PEX syndrome may be associated with a broad spectrum of other ocular, surgical, and systemic complications including cardiovascular and cerebrovascular disease.^6^,^7^

Although the specific pathogenesis of PEX syndrome and associated glaucoma remains unknown, knowledge of the molecular pathology of this fibrotic matrix process has been significantly advanced in recent years at the genome, the transcriptome, and the proteome levels. Collectively, these data support the pathogenetic concept of PEX syndrome as a type of stress-induced elastosis associated with the excessive production and abnormal cross-linking of elastic microfibrils into fibrillar PEX aggregates accumulating in intra- and extraocular tissues.^8^ Pro-fibrotic growth factors, particularly transforming growth factor (TGF)-ß1, and a proteolytic imbalance between matrix metalloproteinases (MMPs) and their tissue inhibitors (TIMPs) appear to contribute to the progressive accumulation of PEX material.

Differential gene expression analyses revealed several classes of genes that may be important in the pathologic production of PEX material.^9^ These genes were mainly related to extracellular matrix metabolism, e.g. *FBN1* (fibrillin-1), *TIMP1* and *TIMP2* (tissue inhibitors of matrix metalloproteinase 1 and 2), *TGF-ß1* (transforming growth factor-ß1) as well as to cellular stress response and regulation, e.g. *ADORA3* (adenosine receptor A3), *CLU* (clusterin), and *mGST-1* (microsomal glutathione-S transferase 1). Additionally, there is increasing evidence that cellular stress conditions, such as oxidative stress and ischemia/hypoxia, as well as low-grade chronic inflammatory processes constitute major mechanisms involved in the pathobiology of PEX syndrome.^10^ Combined with markedly weakened cytoptotective mechanisms, including antioxidant defense, proteasome function, endoplasmic reticulum-related stress response, and DNA repair, these stress conditions may be fibrogenic triggering factors inducing tissue fibrosis.^11^ Indirect immunohistochemical and direct analysis with mass spectrometry have confirmed that PEX material contains mainly elastic proteins, such as elastin, tropoelastin, amyloid P, vitronectin, fibrillin-1, microfibril-associated glycoprotein (MAGP-1), and latent transforming growth factor-ß binding proteins (LTBP-1 and LTBP-2), in addition to proteoglycans, MMPs and TIMPs, cross-linking enzymes including transglutaminase 2, complement factors, apolipoproteins, and the extracellular chaperone clusterin.^12^,^13^

Several lines of evidence, including regional clustering, familial aggregation, and genetic linkage analyses, have previously supported a genetic predisposition to PEX.^14^,^15^ Both population-based and pedigree-based studies have suggested that PEX syndrome is inherited as an autosomal dominant trait with late onset and incomplete penetrance.^16^ A simple inheritance model was not evident suggesting a complex inheritance pattern caused by the contributions of multiple genetic factors and/or environmental conditions.^17^ Accordingly, several chromosomal regions have been tentatively associated with PEX, including the putative gene loci 2p16, 2q35-36, and 3q13-q21 (Wiggs JL. ARVO Meeting 1998, Abstract; Sotirova V. ARVO Meeting 1999, Abstract; Aragon-Martin JA. ARVO Meeting 2005, Abstract) as well as the loci 18q12.1-21.33, 2q, 17p, and 19q, which were identified by a genome-wide scan of 1000 microsatellite markers in a Finnish family.^18^ Recently, genetic studies have demonstrated a highly significant association between PEX and sequence variants in the gene coding for lysyl oxidase-like 1 (*LOXL1*).^19^ Indeed, given the low prevalence of known glaucoma genes, *LOXL1* is currently the most significant genetic risk factor for glaucoma in general.^17^ *LOXL1* is a key enzyme involved in elastic fiber synthesis and homeostasis supporting a role of elastogenesis and elastosis in the pathophysiology of PEX syndrome. This review provides an overview on the recent advances in genetics and genomics of PEX syndrome with a particular focus on the relationship between *LOXL1* and the molecular pathophysiology of PEX syndrome.

## *LOXL1* GENE POLYMORPHISMS AND THEIR IMPLICATIONS IN PEX PATHOPHYSIOLOGY

### Association between *LOXL1* and PEX syndrome/glaucoma

Recent genetic studies in multiple populations have identified the *LOXL1* gene as a major contributor to the risk of developing PEX.^20^ Performing a genome-wide association study, Thorleifsson and co-workers^19^ first detected three common sequence variants or single nucleotide polymorphisms (SNPs) in the *LOXL1* gene on chromosome 15q24.1 that were strongly associated with both PEX syndrome and PEX glaucoma, but not with POAG, in Scandinavians from Iceland and Sweden. The SNPs comprised one intronic SNP, rs2165241 located in intron 1, and two non-synonymous coding SNPs, rs1048661 (R141L) and rs3825942, (G153D) located in exon 1 of *LOXL1*.^19^ A high-risk haplotype (G-G) formed by the two coding SNPs increased the risk for PEX by factor of 27.^19^ Individuals carrying two copies of this high risk haplotype would have a 700 times increased risk of developing PEX than those carrying the low-risk haplotype. However, compared with the general population, the risk of developing PEX is about 2.5-fold only, because approximately 25% of the unaffected controls were also found to carry the high risk haplotype in the homozygous state.

Following this discovery, multiple replication studies in populations from the United States,^21^–^25^ Australia,^26^ Europe,^27^–^30^ Japan,^31^–^36^ China,^37^,^38^ and India^39^ confirmed genetic susceptibility of *LOXL1* polymorphisms to PEX syndrome/glaucoma and verified the *LOXL1* gene as a principal genetic risk factor for this condition worldwide accounting for almost all PEX cases. There were no significant differences between PEX syndrome and PEX glaucoma suggesting that the *LOXL1* gene may contribute to disease onset rather than to IOP elevation and subsequent glaucoma. In support of this observation, no association has been reported with any other type of glaucoma, including POAG, normal tension glaucoma, pigmentary glaucoma, or angle-closure glaucoma. Although SNP rs3825942 (G153D) of *LOXL1* was reported to show weak association with spontaneous cervical artery dissection,^40^ the frequency of risk alleles in both exonic SNPs did not differ between PEX patients with and without cardiovascular disease in a Hungarian population.^30^

In most study populations, the G allele of SNP rs3825942 (G153D) has been reported to be the primary risk-associated variant accounting for 95-100% of PEX cases with an average odds ratio (OR) of 10.89, whereas SNP rs1048661 (R141L) showed a different allele frequency in Japanese and Chinese patients with PEX or was not significantly associated with PEX in other populations.^20^ However, the disease-associated variants are common and show a relatively high prevalence - up to 88% - in the normal population. Consistently, for rs3825942 the sensitivity of the G allele was reported to be 100% but the specificity only 3%, whereas for rs1048661 the sensitivity of the G allele was 95.7% and the specificity 13%.^41^ Therefore, genetic testing for *LOXL1* risk variants may be associated with a very high sensitivity but also a very low specificity making genetic testing of limited use.^41^ These data indicate that in addition to the *LOXL1* risk alleles, other genetic variants or environmental factors may contribute to the risk of developing the PEX phenotype.

To further complicate this issue, a large number of coding variants was recently identified in a black South African population of PEX glaucoma subjects.^42^ Most surprisingly, the A allele of the major susceptibility SNP rs3825942 was the risk allele, which is in sharp contrast to the G allele consistently reported in all other populations. The risk allele G of SNP rs1048661 was however similar to other populations. This hitherto unique observation argues against a causal role of rs3825942 in PEX pathophysiology and suggests that other as yet unknown causal variants of *LOXL1*, e.g. affecting its promoter or other regulatory regions, may contribute to the genetic risk of PEX syndrome/glaucoma.

### Functional implications of *LOXL1* for PEX pathophysiology

*LOXL1* is a member of the lysyl oxidase family of enzymes, copper-dependent amine oxidases that catalyze the covalent cross-linking of collagen and elastin in connective tissues through oxidative deamination of lysine or hydroxylysine side chains.^43^ They comprise five characterized members: lysyl oxidase (LOX) and lysyl oxidase-like 1 to 4 (*LOXL1*-4). *LOXL1* seems to be specifically required for tropoelastin cross-linking and has been shown to be involved in elastic fiber formation, maintenance, and remodeling and to prevent age-related loss of tissue elasticity.^44^ In order to fulfill the cross-linking function, *LOXL1* pro-peptide is selectively targeted to elastic microfibrils at sites of elasto-genesis by binding to both tropoelastin and fibulin-5. Following attachment to the scaffolding structure, the pro-peptide is cleaved off by the endo-metalloproteinase procollagen-C-terminal proteinase (bone morpho-genetic protein 1) for catalytic activation of the enzyme. Deamination of lysine residues causes spontaneous cross-linking of tropoelastin monomers and formation of elastin polymers. Mice deficient in *LOXL1* exhibit massive elastic fiber defects resulting in pelvic organ prolapse, enlarged pulmonary air spaces, vascular abnormalities, and increased laxity of the skin.^44^

Both disease-associated coding SNPs of *LOXL1* reside in exon 1, which encodes the unique N-terminal domain that is required both for proper enzyme activation and for substrate recognition and binding.^45^ Abnormalities were not observed in the highly conserved C- terminal of the protein where the catalytic domains are located. The risk allele G of SNP rs1048661 (R141L) was associated with reduced ocular *LOXL1* expression levels resulting in a 40% decrease in expression compared with normal controls.^46^ In contrast, the risk allele G of rs3825942 (G153D), which was shown to confer the greater risk for PEX in the majority of populations, had no effect on *LOXL1* expression levels. This SNP leads to an amino acid exchange from glycine to aspartic acid at position 153. Although the biological effects of this substitution remain unexplained, genetic programs predict functional consequences on enzyme activation and/or substrate targeting and binding.^40^ In PEX tissues, expression of *LOXL1* was also found to be markedly dysregulated, and this dysregulation clearly depended on the stage of the fibrotic process.^46^ The available data indicate that *LOXL1* is transiently upregulated and activated at early stages of PEX fibrogenesis together with matrix components required for elastic fiber formation, such as tropoelastin, fibrillin-1, and fibulin-4, and participates in the formation of the aberrant fibrillar aggregates accumulating in tissues of PEX patients. Hence, *LOXL1* was found to be a prominent component of fibrillar PEX aggregates in all intra- and extraocular locations, where it co-localized with elastic fiber constituents, particularly fibrillin-1, but not with its normal binding partner fibulin-5, suggesting a shift in substrate specificity for *LOXL1* at sites of pathological matrix formation (Schlötzer-Schrehardt, ARVO Meeting 2009, Abstract). As fibrillin-1 is a major component of PEX fibrils,^47^ it is reasonable to speculate that SNP rs3825942 (G153D) of *LOXL1* is involved in the abnormal processing, assembly, cross-linking, and aggregation of fibrillin-containing microfibrils into mature PEX fibrils.

In later stages of the disease, irrespective of the presence of glaucoma, *LOXL1* expression is decreased below normal homeostatic levels required for elastin maintenance and stability.^48^ The resulting inadequate tissue levels of *LOXL1* may in turn adversely affect elastin metabolism and lead to elastotic alterations, which have been previously described in tissues, such as lamina cribrosa, of patients with advanced PEX.^49^ In fact, lamina cribrosa tissue of PEX eyes reveals a disorganized elastic fiber network and a significant downregulation of *LOXL1* in lamina cribrosa cells together with a reduction of elastic fiber proteins and elastin-specific desmosine cross-links (Schlötzer-Schrehardt, ARVO Meeting 2010, Abstract). These elastotic alterations of laminar beams resulting from *LOXL1* deficiency may have adverse effects on biomechanical properties of this critical structure and may predispose to glaucoma development in eyes with PEX syndrome.

### Additional genetic risk factors for PEX syndrome/glaucoma

In view of the complex inheritance of PEX syndrome, it must be assumed that additional genetic (modifying genes) and/or environmental (fibrotic triggers) factors influence the manifestation of the disease. To identify additional genetic risk factors for PEX syndrome/glaucoma, genome-wide association studies and analyses of sequence variations in candidate genes have been performed but have often achieved conflicting results among the different populations that were studied.

## FUNCTIONAL CANDIDATE GENES FOR PEX SYNDROME

Genetic analysis of several functional candidate genes involved in PEX material formation, encoding fibrillin-1 (*FBN1*), latent TGF-ß binding protein 2 (LTBP2), microfibril-associated protein 2 (MFAP2), transglutaminase 2 (TGM2), transforming growth factor ß1 (*TGF-ß1*), and clusterin (*CLU*), demonstrated significant association of PEX with only one intronic SNP of the clusterin (*CLU*) gene in two independent German cohorts, but not in Italian patients.^50^ Different SNPs and haplotypes of *CLU* have been also nominally associated with PEX in Australian patients.^51^ Clusterin acts as an extracellular chaperone, preventing the precipitation and aggregation of misfolded extracellular proteins. Previous studies demonstrated a significantly reduced expression of clusterin in anterior segment tissues and aqueous humor of PEX eyes, which has been suggested to promote the stable accumulation of abnormally aggregated PEX material in the extracellular space.^52^ These data indicate that common variants in the *CLU* gene do not represent strong genetic modifiers, but may confer some increased risk of developing PEX in certain populations. Another study did not find any association between common polymorphisms of the elastin (*ELN*) gene with PEX syndrome and PEX glaucoma.^53^

Changes in the MMP/TIMP balance and reduced MMP activity in aqueous humor and tissues of PEX eyes represent a major pathogenetic mechanism in abnormal PEX material accumulation.^54^ However, *MMP1* and *MMP3* gene polymorphisms showed no clear-cut significant association with PEX syndrome and PEX glaucoma in Greek patients.^55^ Likewise, polymorphisms in several genes involved in homocysteine metabolism are not associated with PEX syndrome/glaucoma,^56^ although some consider elevated homocysteine levels in plasma, aqueous humor and tear fluid a contribution to PEX pathogenesis and an increased vascular risk in PEX patients.^8^

Performing a genome-wide association study using a DNA pooling approach, Krumbiegel *et al*. (ARVO Meeting 2010, Abstract) identified a significant association of two SNPs in the *CNTNAP2* gene as well as their haplotype with PEX syndrome and PEX glaucoma in German patients. The risk conferred to the disease with an OR of about 1.5, though modest, is typical for many susceptibility variants identified in complex diseases. *CNTNAP2* codes for contactin-associated protein-like 2, a neuronal membrane protein known to regulate various membrane functions and membrane stabilization. Expression and localization patterns of CNTNAP2 protein in ocular tissues, particularly its localization to membranes of cell types involved in PEX material formation, provided further evidence of *CNTNAP2* as an interesting candidate gene for PEX manifestation.

## CANDIDATE GENES FOR GLAUCOMA DEVELOPMENT

Apolipoprotein E (*APOE*) represents a major risk factor for neurodegenerative diseases and previous studies pointed to a possible association between *APOE* alleles and glaucoma in defined populations.^57^ Similarly, the association between *APOE* genotype and PEX glaucoma seems to differ among study populations, indicating a modifying rather than a direct genetic effect. Although Yilmaz *et al*^58^ found the *APOE2* allele to be significantly associated with the development of PEX in a Turkish population, no significant differences in allele and genotype frequencies between PEX and control patients were observed in European patients from Norway^59^ and Germany.^60^

Glutathione S-transferases (GSTs) are a family of enzymes that inactivate xenobiotics and secondary metabolites formed during oxidative stress. Decreased GST function has been suggested by some researchers to exacerbate the direct or indirect damaging effects of oxidative stress on the optic nerve and GST polymorphisms have been suggested to be risk factors for glaucoma development.^61^ Accordingly, *GSTM1* and *GSTT1* polymorphisms have been associated with PEX glaucoma in Saudia Arabian patients.^61^ In contrast, *GSTM1*, *GSTP1*, and *GSTT1* gene polymorphisms were not different between PEX syndrome/glaucoma patients and controls in a Turkish cohort.^62^ Additionally, there was no association between PEX and manganese superoxide dismutase (*Mn-SOD*) polymorphisms in Turkish patients.^63^

Polymorphisms in the *TNFA* gene encoding tumor necrosis factor alpha (TNF-α), one important factor implicated in the pathogenesis of POAG, have been found to be significantly associated with PEX glaucoma in both Pakistani and Iranian populations suggesting a role of immunological factors in PEX-associated neurodegeneration.^64^,^65^ No significant association with *TNFA* polymorphisms was observed in Turkish and European patients.^66^,^67^ Moreover, the distribution of angiotensin-converting enzyme insertion/deletion polymorphisms, which have been previously implicated in the pathogenesis of POAG, was not significantly different in cases with PEX and controls.^68^

No mutations in genes associated with other types of glaucoma or other inherited optic neuropathies, such as *MYOC, OPTN, CYP1B1, WDR36, OPA1*, or *OPA3*, were present in PEX glaucoma patients from Saudi-Arabia.^69^ The same study found only little evidence of mitochondrial DNA mutations in a minority of PEX glaucoma patients (10%).^69^ These findings suggest that typical glaucoma-associated genes and mitochondrial abnormalities are less important determinants of PEX glaucoma than other factors related to the formation and accumulation of PEX material. Another study indicated that the mitochondrial haplotype U is associated with a reduced risk to develop PEX glaucoma in the German population, but further studies are needed to provide more insights into the significance of this association.^70^

## CONCLUSIONS

PEX syndrome is generally considered as a complex, multifactorial, late-onset disease involving a combination of genetic and non-genetic factors in its etiopathogenesis. There is compelling evidence for *LOXL1* as a strong genetic risk factor throughout all populations studied, although the causal variants still need to be characterized. In view of the low penetrance of all disease-associated risk variants identified so far, other genes or environmental factors contribute to the risk of developing the PEX phenotype. The high prevalence of the *LOXL1* variants in the general population may also suggest that there are protective genes and/or environmental factors affecting penetrance. Nevertheless, the effects of the identified *LOXL1* risk variants in the presence of potentially co-modulating external factors, such as TGF-ß1 or oxidative stress, need to be characterized to better understand the functional relevance of *LOXL1* in the pathophysiology of PEX syndrome/glaucoma.

Weaker evidence exists for the contribution of additionally associated genes with relatively small effect sizes only, which seem to differ among study populations indicating a modifying rather than a direct genetic effect. The contributing genes reported may modulate the risk of developing the PEX-specific matrix process, e.g. *CLU* and *CNTNAP2*, or the PEX-associated neurodegeneration, e.g. *APOE*, *GSTs*, and *TNFA*. Several environmental conditions associated with PEX and other fibrotic disorders, including oxidative stress, hypoxia, and elevated levels of pro-fibrotic cytokines (interleukin-6) and growth factors (TGF-ß1), have been shown to regulate both *LOXL1* and clusterin expression as well as synthesis of matrix molecules including elastin and fibrillin-1 *in vitro* (Zenkel *et al*., ARVO Meeting 2009, Abstract) and may therefore act as co-modulating external factors.

Ultimately, both detection and functional characterization of yet unidentified genetic and non-genetic factors contributing to this complex disease may lead to the development of more precise screening tools for individuals at risk for PEX glaucoma. To detect very small genetic effects by multiple additional genes, collaborative studies with large cohorts achieving sufficient power are needed.

## References

[1] Ritch R, Schlötzer-Schrehardt U (2001). Exfoliation syndrome. Surv Ophthalmol.

[2] Ritch R (1994). Exfoliation syndrome: The most common identifiable cause of open-angle glaucoma. J Glaucoma.

[3] Bialasiewicz AA, Wali U, Shenoy R, Al-Saeidi R (2005). Patients with secondary open-angle glaucoma in pseudoexfoliation (PEX) syndrome among a population with high prevalence of PEX: Clinical findings and morphological and surgical characteristics. Ophthalmologe.

[4] Ritch R, Schlötzer-Schrehardt U, Konstas AG (2003). Why is glaucoma associated with exfoliation syndrome?. Progr Ret Eye Res.

[5] Leskea MC, Heijl A, Hyman L, Bengtsson B, Komaroff E (2004). Factors for progression and glaucoma treatment: The Early Manifest Glaucoma Trial. Curr Opin Ophthalmol.

[6] Naumann GO, Schlötzer-Schrehardt U, Küchle M (1998). Pseudoexfoliation syndrome for the comprehensive ophthalmologist. Intraocular and systemic manifestations. Ophthalmology.

[7] Conway RM, Schlötzer-Schrehardt U, Küchle M, Naumann GO (2004). Pseudoexfoliation syndrome: Pathologic manifestations of relevance to intraocular surgery. Clin Exp Ophthalmol.

[8] Schlötzer-Schrehardt U, Naumann GO (2006). Perspective-Ocular and systemic pseudoexfoliation syndrome. Am J Ophthalmol.

[9] Zenkel M, Pöschl E, von der Mark K, Hofmann-Rummelt C, Naumann GO, Kruse FE (2005). Differential gene expression in pseudoexfoliation syndrome. Invest Ophthalmol Vis Sci.

[10] Zenkel M, Lewczuk P, Jünemann A, Kruse FE, Naumann GO, Schlötzer-Schrehardt U (2010). Pro-inflammatory cytokines are involved in initiation of the abnormal matrix process in pseudoexfoliation syndrome/glaucoma. Am J Pathol.

[11] Zenkel M, Kruse FE, Naumann GO, Schlötzer-Schrehardt U (2007). Impaired cellular stress response in eyes with pseudoexfoliation syndrome/glaucoma. Invest Ophthalmol Vis Sci.

[12] Ovodenko B, Rostagno A, Neubert TA, Shetty V, Thomas S, Yang A (2007). Proteomic analysis of exfoliation deposits. Invest Ophthalmol Vis Sci.

[13] Sharma S, Chataway T, Burdon KP, Jonavicius L, Klebe S, Hewitt AW (2009). Identification of *LOXL1* protein and apolipoprotein E as components of surgically isolated pseudoexfoliation material by direct mass spectrometry. Exp Eye Res.

[14] Damji KF, Bains HS, Stefansson E, Loftsdottir M, Sverrisson T, Thorgeirsson E (1998). Is pseudoexfoliation syndrome inherited? A review of genetic and nongenetic factors and a new observation. Ophthalmic Genet.

[15] Orr AC, Robitaille JM, Price PA, Hamilton JR, Falvey DM, De Saint-Sardos AG (2001). Exfoliation syndrome: Clinical and genetic features. Opthalmic Genet.

[16] Forsman E, Cantor RM, Lu A, Eriksson A, Fellman J, Järvelä I (2007). Exfoliation syndrome: Prevalence and inheritance in a subisolate of the Finnish population. Acta Ophthalmol Scand.

[17] Wiggs JL (2008). Association between *LOXL1* and pseudoexfoliation. Arch Ophthalmol.

[18] Lemmelä S, Forsman E, Sistonen P, Eriksson A, Forsius H, Järvelä I (2007). Genome-wide scan of exfoliation syndrome. Invest Ophthalmol Vis Sci.

[19] Thorleifsson G, Magnusson KP, Sulem P, Walters GB, Gudbjartsson DF, Stefansson H (2007). Common sequence variants in the *LOXL1* gene confer susceptibility to exfoliation glaucoma. Science.

[20] Chen H, Chen LJ, Zhang M, Gong W, Tam PO, Lam DS (2010). Ethnicity-based subgroup meta-analysis of the association of *LOXL1* polymorphisms with glaucoma. Mol Vis.

[21] Fingert JH, Alward WL, Kwon YH, Wang K, Streb LM, Sheffield VC (2007). *LOXL1* mutations are associated with exfoliation syndrome in patients from the Midwestern United States. Am J Ophthalmol.

[22] Aragon-Martin JA, Ritch R, Liebmann J, O’Brien C, Blaaow K, Mercieca F (2008). Evaluation of *LOXL1* gene polymorphisms in exfoliation syndrome and exfoliation glaucoma. Mol Vis.

[23] Challa P, Schmidt S, Liu Y, Qin X, Vann RR, Gonzalez P (2008). Analysis of *LOXL1* polymorphisms in a United States population with pseudoexfoliation glaucoma. Mol Vis.

[24] Fan BJ, Pasquale L, Grosskreutz CL, Rhee D, Chen T, DeAngelis MM (2008). DNA sequence variants in the *LOXL1* gene are associated with pseudoexfoliation glaucoma in a U.S. clinicbased population with broad ethnic diversity. BMC Med Genet.

[25] Yang X, Zabriskie NA, Hau VS, Chen H, Tong Z, Gibbs D (2008). Genetic association of *LOXL1* gene variants and exfoliation glaucoma in a Utah cohort. Cell Cycle.

[26] Hewitt AW, Sharma S, Burdon KP, Wang JJ, Baird PN, Dimasi DP (2008). Ancestral *LOXL1* variants are associated with pseudoexfoliation in Caucasian Australians but with markedly lower penetrance than in Nordic people. Hum Mol Genet.

[27] Mossböck G, Renner W, Faschinger C, Schmut O, Wedrich A, Weger M (2008). Lysyl oxidase-like protein 1 (*LOXL1*) gene polymorphisms and exfoliation glaucoma in a Central European population. Mol Vis.

[28] Pasutto F, Krumbiegel M, Mardin CY, Paoli D, Lämmer R, Weber BH (2008). Association of *LOXL1* common sequence variants in German and Italian patients with pseudoexfoliation syndrome and pseudoexfoliation glaucoma. Invest Ophthalmol Vis Sci.

[29] Lemmelä S, Forsman E, Onkamo P, Nurmi H, Laivuori H, Kivelä T (2009). Association of *LOXL1* gene with Finnish exfoliation syndrome patients. J Hum Gen.

[30] Holló G, Gál A, Kóthy P, Molnár JM (2000). *LOXL1* gene sequence variants and vascular disease in exfoliation syndrome and exfoliative glaucoma. J Glaucoma.

[31] Fuse N, Miyazawa A, Nakazawa T, Mengkegale M, Otomo T, Nishida K (2008). Evaluation of *LOXL1* polymorphisms in eyes with exfoliation glaucoma in Japanese. Mol Vis.

[32] Hayashi H, Gotoh N, Ueda Y, Nakanishi H, Yoshimura N (2008). Lysyl oxidase-like 1 polymorphisms and exfoliation syndrome in the Japanese population. Am J Ophthalmol.

[33] Mabuchi F, Sakurada Y, Kashiwagi K, Yamagata Z, Iijima H, Tsukahara S (2008). Lysyl oxidase-like 1 gene polymorphisms in Japanese patients with primary open angle glaucoma and exfoliation syndrome. Mol Vis.

[34] Mori K, Imai K, Matsuda A, Ikeda Y, Naruse S, Hitora-Takeshita H (2008). *LOXL1* genetic polymorphisms are associated with exfoliation glaucoma in the Japanese population. Mol Vis.

[35] Ozaki M, Lee KY, Vithana EN, Yong VH, Thalamuthu A, Mizoguchi T (2008). Association of *LOXL1* gene polymorphisms with pseudoexfoliation in the Japanese. Invest Ophthalmol Vis Sci.

[36] Tanito M, Minami M, Akahori M, Kaidzu S, Takai Y, Ohira A (2008). *LOXL1* variants in elderly Japanese with exfoliation syndrome/glaucoma, primary open-angle glaucoma, normal tension glaucoma, and cataract. Mol Vis.

[37] Chen L, Jia L, Wang N, Tang G, Zhang C, Fan S (2009). Evaluation of *LOXL1* polymorphisms in exfoliation syndrome in a Chinese population. Mol Vis.

[38] Lee KY, Ho SL, Thalamuthu A, Venkatraman A, Venkataraman D, Pek DC (2009). Association of *LOXL1* polymorphisms with pseudoexfoliation in the Chinese. Mol Vis.

[39] Ramprasad VL, George R, Soumittra N, Sharmila F, Vijaya L, Kumaramanickavel G (2008). Association of non-synonymous single nucleotide polymorphisms in the *LOXL1* gene with pseudoexfoliation syndrome in India. Mol Vis.

[40] Kuhlenbäumer G, Friedrichs F, Kis B, Berlit P, Maintz D, Nassenstein I (2007). Association between single nucleotide polymorphisms in the lysyl oxidase-like 1 gene and spontaneous cervical artery dissection. Cerebrovasc Dis.

[41] Challa P (2009). Genetics of pseudoexfoliation syndrome. Curr Opin Ophthalmol.

[42] Williams SE, Whigham BT, Liu Y, Carmichael TR, Qin X, Schmidt S (2010). Major *LOXL1* risk allele is reversed in exfoliation glaucoma in a black South African population. Mol Vis.

[43] Csiszar K (2001). Lysyl oxidases: A novel multifunctional amine oxidase family. Prog Nucleic Acid Res Mol Biol.

[44] Liu X, Zhao Y, Gao J, Pawlyk B, Starcher B, Spencer JA (2004). Elastic fiber homeostasis requires lysyl oxidase-like 1 protein. Nat Genet.

[45] Thomassin L, Werneck CC, Broekelmann TJ, Gleyzal C, Hornstra IK, Mecham RP (2005). The pro-regions of lysyl oxidase and lysyl oxidase-like 1 are required for deposition onto elastic fibers. J Biol Chem.

[46] Schlötzer-Schrehardt U, Pasutto F, Sommer P, Hornstra I, Kruse FE, Naumann GO (2008). Genotype-correlated expression of *LOXL1* in ocular tissues of patients with pseudoexfoliation syndrome/glaucoma and normal subjects. Am J Pathol.

[47] Schlötzer-Schrehardt U, von der Mark K, Sakai LY, Naumann GO (1997). Increased extracellular deposition of fibrillin-containing fibrils in pseudoexfoliation syndrome. Invest Ophthalmol Vis Sci.

[48] Schlötzer-Schrehardt U (2009). Molecular pathology of pseudoexfoliation syndrome/glaucoma – New insights from *LOXL1* gene associations. Exp Eye Res.

[49] Netland PA, Ye H, Streeten BW, Hernandez MR (1995). Elastosis of the lamina cribrosa in pseudoexfoliation syndrome with glaucoma. Ophthalmology.

[50] Krumbiegel M, Pasutto F, Mardin CY, Weisschuh N, Paoli D, Gramer E (2009). Exploring functional candidate genes for genetic association in German patients with pseudoexfoliation syndrome and pseudoexfoliation glaucoma. Invest Ophthalmol Vis Sci.

[51] Burdon KP, Sharma S, Hewitt AW, McMellon AE, Wang JJ, Mackey DA (2008). Genetic analysis of the clusterin gene in pseudoexfoliation syndrome. Mol Vis.

[52] Zenkel M, Kruse FE, Jünemann AG, Naumann GO, Schlötzer-Schrehardt U (2006). Deficiency of the extracellular chaperone clusterin in eyes with pseudoexfoliation syndrome may be implicated in the aggregation and deposition of pseudoexfoliative material. Invest Ophthalmol Vis Sci.

[53] Fan BJ, Figuieredo S, Pasquale LR, Grosskreutz CL, Rhee DJ, Chen TC (2010). Lack of association of polymorphisms in elastin with pseudoexfoliation syndrome and glaucoma. J Glaucoma.

[54] Schlötzer-Schrehardt U, Lommatzsch J, Küchle M, Konstas AG, Naumann GO (2003). Matrix metalloproteinases and their inhibitors in aqueous humor of patients with pseudoexfoliation syndrome, pseudoexfoliation glaucoma, and primary open-angle glaucoma. Invest Ophthalmol Vis Sci.

[55] Tsironi EE, Pefkianaki M, Tsezou A, Kotoula MG, Dardiotis E, Almpanidou P (2009). Evaluation of *MMP1* and *MMP3* gene polymorphisms in exfoliation syndrome and exfoliation glaucoma. Mol Vis.

[56] Fan BJ, Chen T, Grosskreutz C, Pasquale L, Rhee D, DelBono E (2008). Lack of association of polymorphisms in homocysteine metabolism genes with pseudoexfoliation syndrome and glaucoma. Mol Vis.

[57] Al-Dabbagh NM, Al-Dohayan N, Arfin M, Tariq M (2009). Apolipoprotein E polymorphisms and primary glaucoma in Saudis. Mol Vis.

[58] Yilmaz A, Tamer L, Ates NA, Camdeviren H, Degirmenci U (2005). Effects of apolipoprotein E genotypes on the development of exfoliation syndrome. Exp Eye Res.

[59] Ritland JS, Utheim TP, Utheim OA, Espeseth T, Lydersen S, Semb SO (2007). Effects of APOE and CHRNA4 genotypes on retinal nerve fibre layer thickness at the optic disc and on risk for developing exfoliation syndrome. Acta Ophthalmol Scand.

[60] Krumbiegel M, Pasutto F, Mardin CY, Weisschuh N, Paoli D, Gramer E (2010). Apolipoprotein E genotypes in pseudoexfoliation syndrome and pseudoexfoliation glaucoma. J Glaucoma.

[61] Abu-Amero KK, Morales J, Mohamed GH, Osman MN, Bosley TM (2008). Glutathione S-transferase M1 and T1 polymorphisms in Arab glaucoma patients. Mol Vis.

[62] Yilmaz A, Tamer L, Ates NA, Yildirim O, Yildirim H, Atik U (2005). Is GST gene polymorphism a risk factor in developing exfoliation syndrome?. Curr Eye Res.

[63] Yuce H, Hepsen IF, Tekedereli I, Keskin U, Elyas H, Akyol O (2007). Lack of association between pseudoexfoliation syndrome and manganese superoxide dismutase polymorphism. Curr Eye Res.

[64] Razeghinejad MR, Rahat F, Kamali-Sarvestani E (2009). Association of TNFA-308 G/A and TNFRI +36 A/G gene polymorphisms with glaucoma. Ophthalmic Res.

[65] Khan MI, Micheal S, Rana N, Akhtar F, den Hollander AI, Ahmed A (2009). Association of tumor necrosis factor alpha gene polymorphism G-308A with pseudoexfoliative glaucoma in the Pakistani population. Mol Vis.

[66] Tekeli O, Turacli ME, Egin Y, Akar N, Elhan AH (2008). Tumor necrosis factor alpha-308 gene polymorphism and pseudoexfoliation glaucoma. Mol Vis.

[67] Mossböck G, Renner W, El-Shabrawi Y, Faschinger C, Schmut O, Wedrich A (2009). TNF-α-308G>A and -238G>A polymorphisms are not major risk factors in Caucasian patients with exfoliation glaucoma. Mol Vis.

[68] Tekeli O, Turacli ME, Altinok B, Akar N, Elhan AH (2008). No relation between angiotensin-converting enzyme gene polymorphism and pseudoexfoliation. Ophthalm Res.

[69] Abu-Amero KK, Bosley TM, Morales J (2008). Analysis of nuclear and mitochondrial genes in patients with pseudoexfoliation glaucoma. Mol Vis.

[70] Wolf C, Gramer E, Müller-Myhsok B, Pasutto F, Wissinger B, Weisschuh N (2010). Mitochondrial haplogroup U is associated with a reduced risk to develop exfoliation glaucoma in the German population. BMC Genet.

